# Supplementary material for: Preclinical safety study of a combined therapeutic bone wound dressing for osteoarticular regeneration
Source: Nat Commun. 2019 May 14;10:2156. doi: 10.1038/s41467-019-10165-5 (PMC6517440; doi:10.1038/s41467-019-10165-5)
Supplement: Supplementary file 1 — Supplementary Information [file 41467_2019_10165_MOESM1_ESM.pdf]

Preclinical safety study of a combined therapeutic bone wound dressing for osteoarticular regeneration, Keller et al.

Supplemental material

Supplemental table 1. Sheep/treatments included in the safety evaluation step 3

Supplemental table 2. ICRS II parameters for the evaluation of OAR within the implant site

Supplemental table 3. Parameters for the evaluation of tissues adjacent to implant site

Supplemental table 4. Quality control and release criteria for the transplantation of hMSCs

Supplemental table 1. Sheep/treatments included in the safety evaluation step 3

	**Sheep**	**Right proximal**	**Right Distal**	**Left Proximal**	**Left Distal**
**12 weeks**	1	ARTiCAR			ARTiCAR
	2			ARTiCAR	
	3		ARTiCAR		
	4		NT	AG	
	5	NT	AG		NT
	6		NT	AG	
	7	NT		AG	NT
**26 weeks**	1		ARTiCAR		
	2	ARTiCAR			ARTiCAR
	3			ARTiCAR	
	4		ARTiCAR		
	5		NT	AG	
	6	NT	AG		NT
	7	AG		NT	AG
	8		NT	AG	
	9	NT		AG	NT

A total of 16 sheep were implicated in this study. After inducing defects on medial femoral condyles (right or left, distal or proximal), ARTiCAR combined ATMPs was applied and evaluated either at 12 (n = 4-7) or at 26 weeks (n = 4-7), and was compared to autograft treatment (n = 4 at 12 weeks; n = 6 at 26 weeks) or to no treatment (n = 6 at 12 weeks; n = 7 at 26 weeks). A total of 7 sheep/14 defects were treated for analyses at 12 weeks and a total of 9 sheep/18 defects were treated for analysis at 26 weeks. (NT: defect without treatment; AG: autograft.

Supplemental table 2. ICRS II parameters for the evaluation of OAR within the implant site

ICRSII parameters		ARTiCAR	NT	AG	p-value
**Tissue morphology**	*12W*	46±26	62±30	98±3	0.092
0%: Full-thickness fibers / 100%: Normal cartilage birefringence	*26W*	99±2	96±9	100±0	0.411
**Matrix staining**	*12W*	41±27	55±36	59±25	0.855
0%: No staining / 100%: Full metachromasia	*26W*	96±5	82±25	76±24	0.338
**Cell morphology**	*12W*	55±19	61±32	98±3	0.190
0%: No round/oval cells / 100%: Mostly round/oval cells	*26W*	98±4	96±6	99±2	0.286
**Surface architecture**	*12W*	34±12	36±16	40±18	0.870
0%: Delamination, or major irregularity / 100%: Smooth surface	*26W*	69±22	68±29	61±16	0.464
**Basal integration**	*12W*	43±25	51±33	89±8	0.055
0%: No integration / 100% Complete integration	*26W*	94±8	76±31	93±4	0.386
**Formation of a tidemark**	*12W*	35±24	44±30	80±16	0.096
0%: No calcification front / 100%: Tidemark	*26W*	86±11	69±33	89±4	0.214
**Subchondral bone abnormalities/marrow fibrosis**	*12W*	30±22*	54±30	88±6	0.001
0%: Abnormal / 100%: Normal marrow	*26W*	90±14	77±29	94±6	0.343
**Vascularization (within the repaired tissue)**	*12W*	66±16	80±14	100±0	0.080
0%: Present / 100% Absent	*26W*	100±0	99±2	100±0	0.686
**Surface/superficial assessment**	*12W*	46±11	45±29	50±43	0.852
0%: Total loss or complete disruption / 100%: Resembles intact articular cartilage	*26W*	87±9	80±25	66±16	0.275
**Mid/deep zone assessment**	*12W*	31±18	50±35	63±10	0.318
0% Fibrous tissue / 100% normal hyaline cartilage	*26W*	90±8	76±28	77±12	0.161
**Overall assessment**	*12W*	31±18	48±32	65±11	0.311
0%: Bad (fibrous tissue) / 100% Good (hyaline cartilage)	*26W*	88±8	77±27	72±13	0.139

After either 12 or 26 weeks from implant, safety parameters were evaluated for ARTiCAR (n = 4-7 at 12 weeks; n = 4-7 at 26 weeks) and compared to autograft (AG; n = 4 at 12 weeks; n = 6 at 26 weeks) treatment (comparable to mosaicplasty currently performed for cartilage treatment in surgery) and to no treatment (NT; n = 6 at 12 weeks; n = 7 at 26 weeks). Values are represented as mean ± SD. Differences were evaluated with One-Way ANOVA/Kruskal-Wallis test. * = $p \leq 0.05$ between ARTiCAR and autograft.

Supplemental table 3. Parameters for the evaluation of tissues adjacent to implant site

Histological cells evaluation for Cell Type/Response		ARTiCAR	NT	AG
**Polymorphonuclear cells**<br>0: 0 / 1: Rare, 1-5/phf* / 2: 6-10/phf* / 3: Heavy infiltrate / 4: Packed	*12W*<br>*26W*	0.00 ± 0.00<br>0.00 ± 0.00	0.00 ± 0.00<br>0.00 ± 0.00	0.00 ± 0.00<br>0.00 ± 0.00
**Lymphocytes**<br>0: 0 / 1: Rare, 1-5/phf* / 2: 6-10/phf* / 3: Heavy infiltrate / 4: Packed	*12W*<br>*26W*	1.00 ± 0.00<br>0.20 ± 0.45	1.00 ± 0.00<br>0.14 ± 0.38	0.00 ± 0.00<br>0.60 ± 0.55
**Plasma cells**<br>0: 0 / 1: Rare, 1-5/phf* / 2: 6-10/phf* / 3: Heavy infiltrate / 4: Packed	*12W*<br>*26W*	0.50 ± 0.58<br>0.20 ± 0.45	0.00 ± 0.00<br>0.29 ± 0.49	0.00 ± 0.00<br>0.20 ± 0.45
**Macrophages**<br>0: 0 / 1: Rare, 1-5/phf* / 2: 6-10/phf* / 3: Heavy infiltrate / 4: Packed	*12W*<br>*26W*	1.00 ± 0.00<br>0.60 ± 0.55	1.00 ± 0.00<br>0.57 ± 0.53	1.00 ± 0.00<br>1.00 ± 0.00
**Giant Cells**<br>0: 0 / 1: Rare, 1-5/phf* / 2: 6-10/phf* / 3: Heavy infiltrate / 4: Packed	*12W*<br>*26W*	1.00 ± 0.00<br>1.00 ± 0.00	0.00 ± 0.00<br>0.14 ± 0.38	0.00 ± 0.00<br>0.40 ± 0.55
**Necrosis/Tissue Degeneration**<br>0: 0 / 1: Slight / 2: Moderate / 3: Marked / 4: Severe	*12W*<br>*26W*	2.50 ± 0.58<br>1.20 ± 1.3	1.83 ± 0.75<br>1.00 ± 1.00	1.50 ± 0.58<br>1.40 ± 1.14
**Infection**<br>0: 0 / 1: Slight / 2: Moderate / 3: Marked / 4: Severe	*12W*<br>*26W*	0.00 ± 0.00<br>0.00 ± 0.00	0.00 ± 0.00<br>0.00 ± 0.00	0.00 ± 0.00<br>0.00 ± 0.00
**Fibrinous exudate**<br>0: 0 / 1: Slight / 2: Moderate / 3: Marked / 4: Severe	*12W*<br>*26W*	0.00 ± 0.00<br>0.00 ± 0.00	0.00 ± 0.00<br>0.00 ± 0.00	0.00 ± 0.00<br>0.00 ± 0.00
**Neovascularisation**<br>0: 0 / 1: Minimal (1-3 capillary buds) / 2: Groups of 4-7 capillaries with supporting fibroblastic structures / 3: Broad band of capillaries with supporting structures/ 4: Extensive band of capillaries with supporting fibroblastic structures	*12W*<br>*26W*	2.25± 0.50<br>1.20 ± 1.10	1.50 ± 1.84<br>0.29 ± 0.49	1.00 ± 0.00<br>0.80 ±0.84
**Fatty infiltrate**<br>0: 0 / 1: Minimal amount of fat associated with fibrosis / 2: Several layers of fat and fibrosis / 3: Elongated and broad accumulation of fat cells about the implant site / 4: Extensive fat completely surrounding the implant	*12W*<br>*26W*	0.00 ± 0.00<br>0.00 ± 0.00	0.00 ± 0.00<br>0.00 ± 0.00	0.00 ± 0.00<br>0.00 ± 0.00
**Fibrocytes/fibroconnective tissue, fibrosis**<br>0: 0 / 1: Narrow band / 2: Moderately thick band / 3: Thick band / 4: Extensive band	*12W*<br>*26W*	2.75± 0.50<br>1.60 ± 1.14	1.83 ± 0.98<br>0.71 ± 0.76	1.50 ± 0.58<br>1.20 ± 1.10
**Cartilage and/or cartilage fibrocartilage formation deeper than the defect**<br>0: 0 / 1: Slight / 2: Moderate / 3: Marked / 4: Severe	*12W*<br>*26W*	1.25 ± 0.50<br>0.40 ± 0.89	1.67 ± 0.52<br>1.29 ± 0.76	0.75 ± 0.50<br>1.00 ± 0.00
**Approximate depth of tissue reaction**<br>Evaluated in mm	*12W*<br>*26W*	10.50 ± 4<br>7.60 ± 4.51	7.33 ± 1.63<br>8.93 ± 5.25	9.00 ± 2.00<br>9.40 ± 4.16
**Void space underneath defect**<br>P: Present / A: Absent	*12W*<br>*26W*	NA<br>NA	NA<br>NA	NA<br>NA
**Mucocinous material**<br>P: Present / A: Absent	*12W*<br>*26W*	NA<br>NA	NA<br>NA	NA<br>NA
**Hemosiderin-laden macrophages**<br>0: 0 / 1: Slight / 2: Moderate / 3: Marked / 4: Severe	*12W*<br>*26W*	0.50 ± 0.58<br>0.80 ± 0.45	0.67 ± 0.52<br>0.29 ± 0.49	0.50 ± 0.58<br>0.20 ± 0.45

After either 12 or 26 weeks from implant, safety parameters were evaluated for ARTiCAR (n = 4-7 at 12 weeks; n = 4-7 at 26 weeks) and compared to autograft (AG; n = 4 at 12 weeks; n = 6 at week 26) treatment (comparable to mosaicplasty currently performed for cartilage treatment in surgery) and to

no-treatment (NT; n = 6 at week 12; n = 7 at week 26). Values are represented as mean ± SD. NA: not applicable, phf*: per high powered (x400) field.

Supplemental table 4. Quality control and release criteria for the transplantation of hMSCs

		**at seeding (bone marrow sample)**	**at harvesting (MSCs for transplantation)**
**Quality Control**	morphofunctional	cell count<br>cell viability<br>colony-forming ability	cell count<br>cell viability<br>colony-forming ability
	sterility	aerobic microorganisms<br>anaerobic microorganisms<br>mycoplasma	aerobic microorganisms<br>anaerobic microorganisms<br>mycoplasma<br>endotoxins
	immunophenotype	n/a	HLA-I, CD73^{+}, CD90^{+}, CD105^{+}, CD166^{+}, HLA-DR^{-}, CD31^{-}, CD34^{-}, CD45^{-}, CD80^{-}
	multipotency	n/a	adipogenic diff.<br>chondrogenic diff.<br>osteogenic diff.

	**criterion**	**at harvesting (MSCs for transplantation)**
**Release Criteria**	cell count	$\geq 2.4 \times 10^{7}$
	cell viability	≥ 90%
	morphology of the adherent cells	fibroblast-like (spindle shaped)
	CD73, CD90, CD105, CD166	≥ 90% positive cells
	HLA-DR, CD31, CD34, CD45, CD80	< 10% positive cells

The quality control and the minimum release criteria implemented for testing human MSCs before transplantation in human patients (adapted from Dominici et al., 2006).
